# Cundurango at Less Price

**Published:** 1871-10

**Authors:** 


### Cundurango at Less Price.

This drug is now offered in our market at 75 per cent. less than formerly. Wm. H. Peabody receives regular supplies from the "PROPRIETORS," Bliss, Keene & Co., and furnishes it at their prices.

---

NESTLE'S LACTEOUS FARINA, or "Mother's Milk," has been used by some of our leading physicians for the last two years, who speak favorably of it as a substitute for Mother's Milk.

